# Des lésions pseudo-chéloïdiennes révélant une sarcoïdose cutanée et pulmonaire

**DOI:** 10.11604/pamj.2013.14.45.2362

**Published:** 2013-02-01

**Authors:** Fadwa El Amrani, Badredine Hassam

**Affiliations:** 1 Service de Dermatologie, CHU Ibn Sina, Université Med V, Souissi, Rabat, Maroc

**Keywords:** Lésions pseudo-chéloïdiennes, sarcoïdose cutanée, sarcoïdose pulmonaire, érythéme, Pseudo-keloid lesions, cutaneous sarcoidosis, pulmonary sarcoidosis, erythema

## Images en medicine

La sarcoïdose est une affection systémique granulomateuse d’étiologie inconnue. Ses manifestations dermatologiques sont très polymorphes. Elles s’observent approximativement chez 25% des patients atteints de sarcoïdose et peuvent être inaugurales. Le diagnostic de sarcoïdose repose sur un faisceau d’arguments cliniques, histologiques, biologiques et radiologiques. Dans plus de la moitié des cas, l’évolution est spontanément favorable dans un délai de 12 à 36 mois. La corticothérapie constitue le traitement de référence, cependant, d’autres médicaments peuvent aussi être efficaces: les immunosuppresseurs, les antipaludéens de synthèse et l’infliximab. Nous rapportons le cas d’un patient de 48 ans, sans antécédents pathologiques notables, qui consulte pour des lésions cutanées asymptomatiques évoluant depuis un an, sans autres signes associés, notamment respiratoires. L’examen dermatologique notait la présence de lésions papulo-nodulaires, érythémato-violines, fermes, pseudo-chéloïdiennes et bien limitées, siégeant à la face antérieure du thorax, des bras et aux faces latérales du cou. Le reste de l’examen était sans anomalie. La biopsie cutanée a montré un granulome épithélio et giganto-cellulaire sans nécrose caséeuse. L’IDR à la tuberculine était négative, le taux de l’enzyme de conversion de l’angiotensine était élevé et la TDM thoracique a révélé une atteinte pulmonaire de stade III. Les autres explorations, notamment rénale et hépatique n’ont pas révélé d’anomalies. Le diagnostic de sarcoïdose cutanéo-pulmonaire a été retenu. Le patient a été mis sous corticothérapie générale et antipaludéens de synthèse avec une diminution de la taille et du nombre des lésions cutanées après 6 mois de traitement.

**Figure 1:**
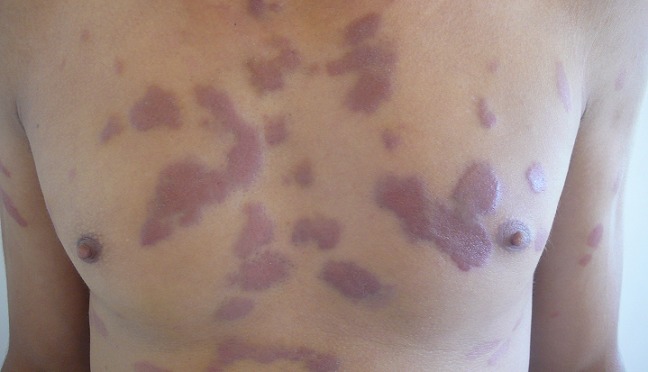
Des lésions papulo-nodulaires, érythémato-violines et pseudo-chéloidiennes, bien limitées de la face antérieure du thorax et des bras

